# Energy, Macronutrients, Dietary Fibre and Salt Intakes in Older Adults in Ireland: Key Sources and Compliance with Recommendations

**DOI:** 10.3390/nu13030876

**Published:** 2021-03-08

**Authors:** Laura Kehoe, Janette Walton, Breige A. McNulty, Anne P. Nugent, Albert Flynn

**Affiliations:** 1 School of Food & Nutritional Sciences, University College Cork, T12 K8AF Cork, Ireland; janette.walton@cit.ie (J.W.); a.flynn@ucc.ie (A.F.); 2 Department of Biological Sciences, Munster Technological University, T12 P928 Cork, Ireland; 3 UCD Institute of Food & Health, University College Dublin, Belfield, D04 V1W8 Dublin, Ireland; breige.mcnulty@ucd.ie (B.A.M.); A.Nugent@qub.ac.uk (A.P.N.); 4 Institute for Global Food Security, Queens University Belfast, Belfast BT7 1NN, UK

**Keywords:** energy, macronutrients, dietary fibre, salt, sources, older adults

## Abstract

The global population is rapidly ageing. Adequate nutritional status can play a key role in preventing or delaying the progression of age-related diseases. The aim of this study was to estimate the usual intake of energy, macronutrients, dietary fibre and salt in order to determine compliance with recommendations and to identify the sources of these nutrients in older adults (≥65 years) in Ireland. This study is based on data from the cohort of older adults aged ≥65 years (*n* = 226) in the Irish National Adult Nutrition Survey (NANS) (2008–2010) which estimated food and nutrient intakes in a representative sample of adults 18–90 years using a 4 day weighed food record. This study found that while intakes of macronutrients are generally sufficient in this population group, older adults in Ireland have high intakes of total fat, saturated fat, sugar and salt and low intakes of dietary fibre. Future strategies to address the nutritional issues identified in older adults could include the promotion of healthy food choices together with improvements of the food supply including reformulation (fat, sugar and salt) to support successful ageing of our population.

## 1. Introduction

The global population is rapidly ageing and it is estimated that by 2050, one in five people will be aged 60 years or over [1]. While most of this population will be in good health, remain fully independent and engaged in their communities, many will be confronted with the ageing process, resulting in a decreased quality of life, illness and disease [2]. Adequate nutritional status can play a key role in preventing or delaying the progression of many of the chronic conditions prevalent among the older adult population such as cardiovascular disease (CVD), diabetes mellitus, hypertension, obesity, reduced cognitive function and osteoporosis [3,4,5,6,7,8,9]. However, due to the practical, physiological and social changes associated with ageing such as changes in food accessibility and cooking abilities, decreased food intake, impaired sensory perception, malabsorption, declining activity and increased disability, older adults have an increased risk of malnutrition or nutrient deficiencies which have been recognised as important predictors of morbidity and mortality [10,11,12].

Research in recent years has highlighted potentially varying nutritional needs among those older adults categorised as ‘young-old’ and ‘old-old’. However, to date, most internationally accepted Dietary Reference Values (DRVs) have been set based on requirements for younger adults and have not distinguished between different categories of older adults [13,14,15,16,17,18]. The Scientific Advisory Committee on Nutrition (SACN) in the UK have recently published a position statement on nutrition and older adults living in the community, including those aged 80 years and older [19]. In Ireland, the most recent food and nutrition policy for older adults was published by the Food Safety Authority of Ireland (FSAI) in 1999 [20] which is currently being updated to provide food-based dietary guidelines to meet nutrition goals for older adults.

Recent studies on community-dwelling older adults across Europe have highlighted suboptimal distributions of macronutrient intakes in older adults, similar to that of their younger adult counterparts with too little energy provided by carbohydrates and protein and too much from fat [21,22,23,24,25,26,27,28,29]. Furthermore, high intakes of saturated fat, sugar and salt and low intakes of dietary fibre have been reported [21,22,23,24,25,26,27,28,29].

The National Adult Nutrition Survey (NANS) (2008–2010) is the most recent nationally representative food consumption survey of adults in the Republic of Ireland and includes those ≥65 years. This study aims to use data from the NANS to estimate the usual intake of energy, macronutrients, dietary fibre and salt, to determine compliance with recommendations and to identify the food groups that are contributing to these nutrients in older adults (≥65 years) in Ireland (supporting the development of the updated food-based dietary guidelines for older adults).

## 2. Materials and Methods

### 2.1. Study Sample

Analyses for the present study are based on data from the Irish National Adult Nutrition Survey (NANS), which was a cross-sectional survey conducted in the Republic of Ireland in the period 2008–2010 by the Irish Universities Nutrition Alliance (IUNA) units at University College Cork and University College Dublin to establish a database of habitual food and beverage consumption in a representative sample of adults aged 18–90 years (*n* = 1500). A detailed survey methodology is available at www.iuna.net (accessed on 1 November 2020) and an overview of the methods relevant to this study is outlined below. For the purposes of this study, data from the subgroup of adults aged ≥65 years (*n* = 226) in the NANS were used.

### 2.2. Ethical Approval

This study was conducted according to the guidelines laid down in the Declaration of Helsinki and ethical approval was obtained from the Clinical Research Ethics Committee of the Cork Teaching Hospitals, University College Cork and the Human Ethics Research Committee of University College Dublin (Ref: ECM 3 (p) 04/11/08). Written informed consent was obtained from all participants.

### 2.3. Sampling and Recruitment Methodology

The fieldwork phase was carried out between October 2008 and April 2010. Eligible participants in the NANS were adults aged 18 years and over who were generally healthy, free living and who were not pregnant or breastfeeding. A sample of adults were randomly selected from a database of names and addresses held by Data Ireland (An Post) and an introductory letter and information leaflet were posted to each person selected from the database. A second level of recruitment was used in which names and addresses were compiled through referrals from participants and participation was invited for those that were contactable. A researcher called to potential respondents’ homes to introduce the survey and invite participation. If the individual agreed to participate, a consent form was signed and the survey commenced. If the person was not at home, the researcher called on three more occasions on different days and at different times, before deeming them ineligible. The final response rate for the survey was 60%. Demographic analysis of the NANS sample has shown it to be nationally representative of adults in the Republic of Ireland with respect to age group, gender, social class and geographical location when compared to Census 2006 data for the Republic of Ireland.

### 2.4. Food Consumption Data and Quantification

Food and beverage intake data were collected using a 4 day semi-weighed food record. For all participants, the study period included at least one weekend day. The researcher made three visits to the participant during the 4 day recording period: an initial training visit to demonstrate how to complete the food diary and use the food scales, a second visit 24–36 h into the recording period to review the diary, check for completeness and clarify details regarding specific food descriptors and quantities, and a final visit 1 or 2 days after the recording period to check the recordings from the final days and to collect the diary. Participants were asked to record detailed information regarding the amount, type and brand of all food, beverages and nutritional supplements consumed over the four day period and where applicable the cooking methods used, details of recipes and any description of any leftovers.

A quantification protocol that had been previously established by the IUNA using a hierarchical approach was adapted for the NANS. Foods and beverages were weighed either by the participant (using a provided portable food scales (Tanita KD-400, Japan) or based on manufacturer weights from the product label. This method was used to quantify 56% of foods and drinks consumed in the NANS. For foods that were not weighed, a photographic food atlas was used to quantify 16% of foods and beverages consumed [30] and standard portion sizes were used to assign weights to a further 15% of foods or beverages [31]. Household measures were used to quantify 11% of food and beverages and estimated quantities (based on the participants other entries) were used to quantify 2% of food and beverage items.

### 2.5. Nutrient Composition of Foods

Dietary intake data were analysed using WISP^©^ (Tinuviel Software, Anglesey, UK), which contains food composition data from McCance and Widdowson’s The Composition of Foods, sixth [32] and fifth [33] editions plus all nine supplemental volumes [34,35,36,37,38,39,40,41,42]. During the NANS, modifications were made to the food composition database to include recipes of composite dishes, nutritional supplements, fortified foods, generic Irish foods that were commonly consumed and new foods on the market [43]. Additionally, the food composition database was updated with values for total fat, saturated fat, monounsaturated fat, polyunsaturated fat, α-linolenic acid (ALA), eicosapentaenoic acid (EPA), docosahexaenoic acid (DHA), free sugar and sodium, the details of which have been outlined in detail elsewhere [44,45,46].

### 2.6. Estimation of Usual Intakes

Distributions of the usual intake of energy and macronutrients were estimated using the validated National Cancer Institute (NCI)-Method [47] using SAS Enterprise Guide^©^ Version 6.1 (SAS Institute Inc., Cary, NC, USA). The NCI-method has been implemented in SAS macros (version 2.1) which were downloaded from the website www.riskfactor.gov/diet/usualintakes/macro.html (date of download: July 2015). For these analyses, the covariates used were age group and gender. Using these macros, distributions of usual intakes were estimated for energy and nutrients for the total population of older adults, by gender and age group (65–74 year olds and ≥75 year olds).

### 2.7. Compliance with Energy and Macronutrient Recommendations

In line with recent calls for harmonisation of dietary data and for appropriate comparison with other studies, nutrient intakes were compared with the most recent DRVs for the general population from the European Food Safety Authority (EFSA) which have been updated and developed over the past 10 years underpinned by the latest scientific evidence [48]. Where appropriate DRVs were not available from the EFSA, the UK SACN or Department of Health (DoH) (1991) DRVs were used as they are the most applicable to the Irish population [49]. In the absence of guidance at an EU level for free sugars, intakes were compared to the WHO and SACN recommendations [50,51]. Salt intakes were compared to the maximum population target of <6 g set by the FSAI which is in line with recommendations from the WHO and country specific guidelines across Europe [49,52,53,54,55,56]. The prevalence of inadequate intake of energy was estimated excluding under-reporters [57] using the estimated average requirements (EAR) from the EFSA for older adults using physical activity levels (PAL) of both 1.4 (sedentary) and 1.6 (moderately active) [58]. However, it should be noted that while the cut-point method has been shown to be effective in obtaining a realistic estimate of the prevalence of dietary inadequacy [59], it may not be appropriate for certain nutrients such as energy as intakes and requirements are highly correlated [60]. Mean intakes for carbohydrate and fat were compared to reference intake ranges recommended by the EFSA for carbohydrate (45–60% total energy (TE)) and for total fat (20–35%TE) [61,62]. Saturated fat intakes were compared to the maximum average population intake recommendation of 10%TE from the UK DoH (1991) [49]. Monounsaturated (MUFA) and polyunsaturated (PUFA) fat intakes were compared to the minimum average population intake recommendations from the UK DoH of 12%TE for MUFA and 6%TE for PUFA [49]. Mean intakes of ALA and EPA and DHA combined were compared to adequate intakes as proposed by the EFSA (ALA: 0.5%TE, EPA + DHA: 250 mg/day) [62]. Mean protein intakes (g/kg body weight) were compared to the average requirement proposed by the EFSA of 0.66 g/kg body weight per day [63]. Free sugar intakes were compared to the World Health Organisation (WHO) recommendation of <10%TE for individuals and the UK SACN recommendation for an average population intake of <5%TE [50,51]. For dietary fibre, mean intakes were compared to the EFSA adequate intake (AI) of 25 g/day [61]. Dietary salt was measured as both sodium equivalents from food sources only (i.e., excluding discretionary salt: added in cooking or at the table) and as urinary sodium excretion (measured from spot urine samples as described previously [64]) and intakes were compared to the maximum population target (<6 g/day) set by the FSAI [55].

### 2.8. Contribution of Food Groups to Intakes of Energy, Macronutrients, Dietary Fibre and Salt

Using SPSS^©^ for Windows™ Version 26.0 (SPSS, Inc., IBM, Chicago, IL, USA), the percent contribution of each food group to mean daily intakes of energy, macronutrients, dietary fibre and salt was calculated by the mean proportion method [65] and the key contributors to each nutrient were reported in order of importance for the total population and by gender. The mean proportion method provides information about the sources that are contributing to the nutrient intake ‘per person’ and is the preferred method when determining important food sources of a nutrient for individuals in a population group as opposed to investigating the sources of a nutrient within the food supply.

### 2.9. Statistical Analysis

Statistical analysis was carried out using SPSS^©^ for Windows™ Version 26.0 (SPSS, Inc., IBM, Chicago, IL, USA). Differences in nutrient intakes across gender and age groups were assessed using Independent Samples T Tests for normally distributed data and Mann–Whitney U Tests where data were not normally distributed. To minimise Type 1 errors (due to multiple testing), intakes were considered to be significantly different from each other if *p* < 0.001.

## 3. Results

Table 1 presents the key characteristics of this cohort of older adults compared with the national population of older adults from the Republic of Ireland (ROI) from Census 2006 [66]. In line with the total population of older adults in Ireland, the total sample of older adults (*n* = 226) in the NANS consisted of 47% men and 53% women, with 66% aged 65–75 years and 34% aged 75+ years. Among this cohort, 54% of data collection took place in the Winter (September-February) and 46% in the Summer (March-August). Twenty percent of older adults in the NANS lived in a rural area, while 80% lived in an urban area, which is a higher proportion of older adults in the NANS living in urban areas compared to the total population in Ireland. Similar to the total population aged 15 years and older in Ireland from the ROI Census, 38% of older adults in the NANS were categorised (based on previous occupation) as professional, managerial & technical workers, 19% were non-manual workers, 21% were skilled manual workers and 11% were semi-skilled/unskilled workers (including students). In terms of education, 30% of older adults had primary education, 23% had intermediate education, 20% had secondary and 27% had tertiary education.

The distribution of the intake of energy, total fat, fatty acids, protein, carbohydrates, sugars, dietary fibre, salt and alcohol are presented for the total population and by gender in Table 2; Table 3 and by age group (65–75 year olds and 75+ year olds) in Supplementary Tables S1 and S2.

Men had significantly higher absolute (g) intakes of all nutrients and energy-adjusted intakes of saturated fat, MUFA, EPA and DHA and lower energy-adjusted intakes of PUFA, ALA, protein, carbohydrate, sugars (total, added and free) and dietary fibre compared to women. These findings indicate that for most nutrients (with the exception of saturated fat, MUFA, EPA and DHA) men do not consume a more nutrient dense diet than women. Men also had significantly higher intakes of alcohol compared to women. No differences in intakes were noted between 65–75 year olds and 75+ year olds for any nutrient examined or for alcohol.

**Figure 1:**
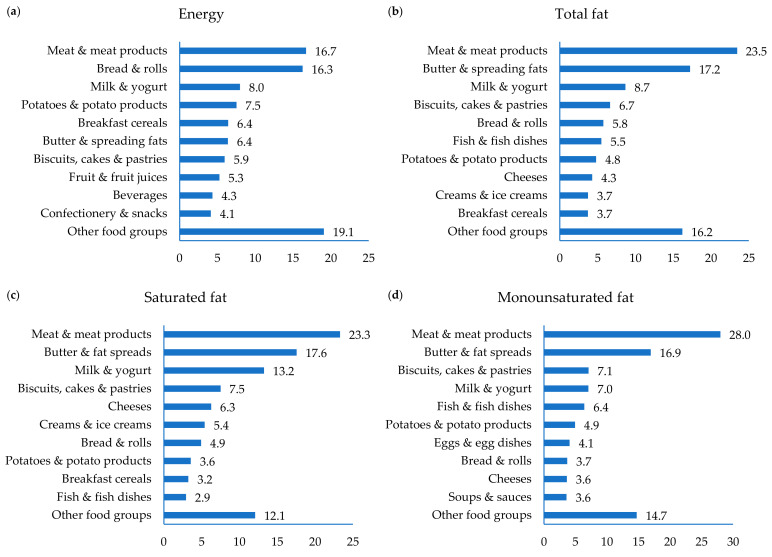

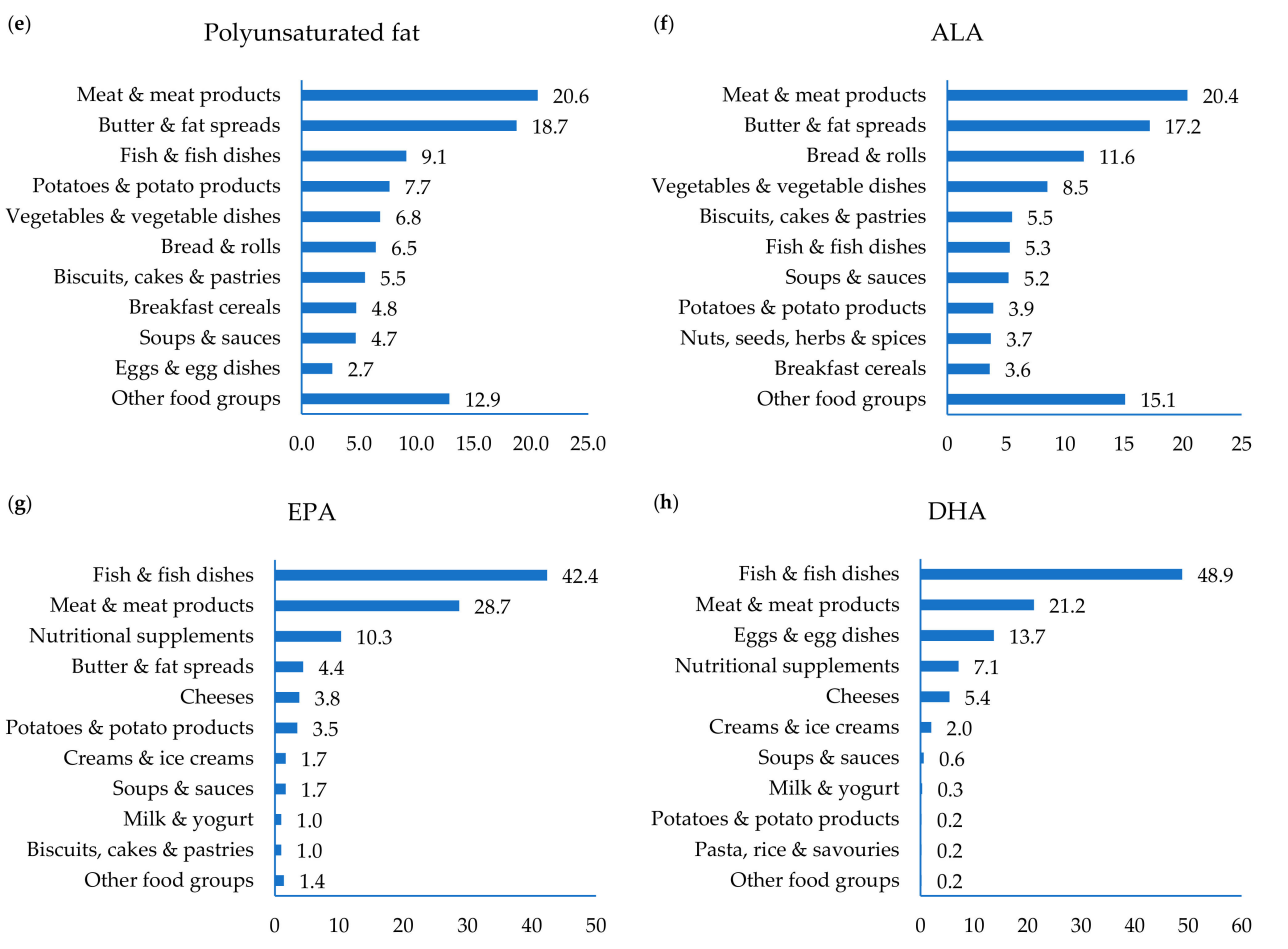
Contribution (%) of food groups to intakes of (**a**) energy, (**b**) total fat, (**c**) saturated fat, (**d**) monounsaturated fat, (**e**) polyunsaturated fat, (**f**) ALA: α-Linolenic acid, (**g**) EPA: eicosapentaenoic acid and (**h**) DHA: docosahexaenoic acid in older adults in Ireland.

The mean intake of energy in older adults in Ireland was 7.4 MJ/day (8.3 MJ/day for men and 6.6 MJ/day for women) (Table 2). One-quarter (25%) of older adults had energy intakes below the age appropriate EAR from the EFSA for a PAL of 1.4 and 44% had energy intakes below the appropriate EAR from the EFSA for a PAL of 1.6 (analysis excluding under-reporters). The key dietary sources of energy were ‘meat & meat products’ (17%), ‘bread & rolls’ (16%), ‘milk & yogurt’ (8%), ‘potatoes & potato products’ (8%) and ‘breakfast cereals’ (6%), providing 55% of intakes (Figure 1). ‘Butter & spreading fats’ and ‘biscuits, cakes & pastries’ each provided a further 6% of energy intake.

The mean intake of total fat among older adults was 68 g/day, providing 34%TE (Table 2). Sixty percent of older adults had mean daily intakes of fat within the recommended intake range of 20–35%TE, with 40% exceeding 35%TE and no individual having intakes below 20%TE. The key sources of total fat in the diet were ‘meat & meat products’ (23%), ‘butter & spreading fats’ (17%), ‘milk & yogurt’ (9%) and ‘biscuit, cakes & pastries’ (7%) (Figure 1).

The mean intake of saturated fat in older adults was 27 g/day (14%TE) (Table 2), which is greater than the recommendation of <10%TE. The key sources of saturated fat were ‘meat & meat products’, ‘butter & spreading fats’ and ‘milk & yogurt’, providing 23, 18 and 13% of intakes, respectively (Figure 1). The mean intake of MUFA was 12%TE, in line with the UK DoH recommendation that MUFA should provide an average of 12%TE. The key sources of MUFA were ‘meat & meat products’ (28%), ‘butter & spreading fats’ (17%), and ‘biscuits, cakes & pastries’ (7%) (Figure 1). The mean intake of PUFA was 6%TE, in line with the UK DoH recommendation that PUFA should provide an average of 6%TE. The primary sources of PUFA in the diet were ‘meat & meat products’, ‘butter & spreading fats’ and ‘fish & fish dishes’, providing 21, 19 and 9% of intakes, respectively (Figure 1).

The mean intake of alpha-linolenic acid (ALA) was 0.56%TE (Table 2), similar to the recommended adequate intake (AI) of 0.5%TE. The key sources of ALA were ‘meat & meat products’, ‘butter & spreading fats’ and ‘bread & rolls’, providing 20, 17 and 12% of intakes, respectively (Figure 1). The mean intake of ecoisapentonic acid (EPA) and docosahexaenoic acid (DHA) (combined) was 602 mg/day, which is well above the AI of 250 mg for adults (Table 2). ‘Fish & fish dishes’ and ‘meat & meat products’ provided almost three-quarters of both EPA and DHA in the diets of older adults in Ireland (71 and 70%, respectively) (Figure 1).

**Figure 2:**
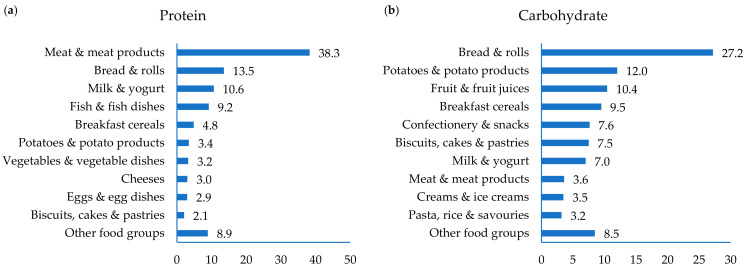

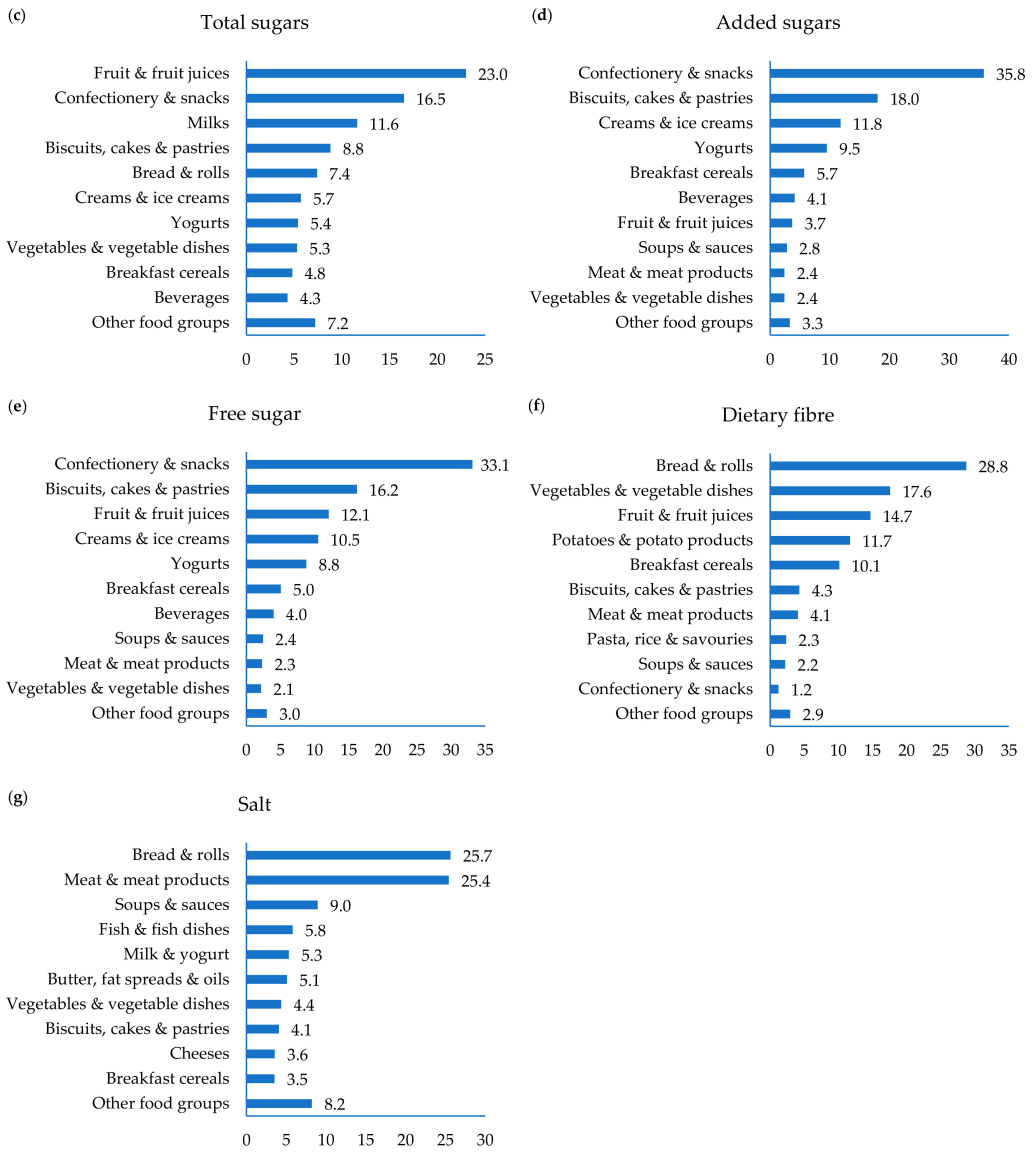
Contribution (%) of food groups to intakes of (**a**) protein, (**b**) carbohydrate, (**c**) total sugars, (**d**) added sugars, (**e**) free sugars, (**f**) dietary fibre and (**g**) salt in older adults in Ireland.

The mean intake of protein was 78 g/day, providing 18%TE (Table 2). The mean intake of protein adjusted for body weight was 1.0 g/kg per day; however, 35% of older adults had intakes of protein below the estimated average requirement (EAR) of 0.66 g/kg/day. ‘Meat & meat products provided 38% of protein intake with ‘breads & rolls’, ‘milk & yogurt’ and ‘fish and fish dishes’, providing a further 14, 11 and 9% of intakes, respectively (Figure 2).

The mean intake of carbohydrates was 208 g/day (44%TE) (Table 3). Over half of older adults (54%) had intakes of carbohydrate below the lower bound reference intake range of 45%TE while no individual had intakes greater than 60%TE. The key sources of protein in the diets of older adults in Ireland were ‘bread & rolls’ (27%), ‘potatoes & potato products’ (12%), ‘fruits & fruit juices’ (10%), breakfast cereals (10%), ‘biscuits, cakes & pastries’ (8%) and ‘confectionary & snacks’ (8%) (Figure 2).

The mean intake of total and added sugars were 86 and 37 g/day, respectively (Table 3). The key sources of total sugars were ‘fruit & fruit juices’, ‘confectionary & snacks’, milks and ‘biscuits, cakes & pastries’, providing 23, 17, 12 and 9% of intakes, respectively and the key sources of added sugars were ‘confectionary & snacks’, ‘biscuits, cakes & pastries’, ‘creams, ice-creams & chilled desserts’ and yogurts, providing 36, 28, 12 and 10% of intakes, respectively (Figure 2). The mean intake of free sugars was 41 g/day (8%TE). This exceeds the UK recommendation of a maximum average population intake of <5%TE. Thirty percent of older adults had intakes of free sugars greater than the WHO recommendation of <10%TE and 76% had intakes greater than the WHO conditional recommendation of <5%TE. The key sources of free sugars in the diet were ‘confectionary & snacks’, ‘biscuits, cakes & pastries’, ‘fruit & fruit juices’, ‘creams, ice-creams & chilled desserts’ and yogurts, providing 33, 16, 12, 11 and 9% of intakes, respectively (Figure 2).

The mean intake of dietary fibre was 19 g/day which is below the AI of 25 g/day (Table 3). The key sources of dietary fibre were ‘bread & rolls’, ‘vegetable & vegetable dishes’, ‘fruit & fruit juices’, ‘potatoes & potato products’ and breakfast cereals, providing 29, 18, 15, 12 and 10% of dietary fibre intakes, respectively (Figure 2).

The mean intake of salt from food sources only (excluding salt added at the table or in cooking) was 5.6 g/day (Table 3). Salt intake from all sources (measured by urinary sodium excretion) was 11.4 g/day for men and 7.2 g/day for women which exceeds the maximum population target of <6 g/day (data not shown). The key sources of salt were ‘bread & rolls’, ‘meat & meat products’, ‘soups & sauces’ and ‘fish & fish dishes’ contributing 26, 25, 9 and 6% of intakes, respectively (Figure 2).

## 4. Discussion

This study provides information on energy, macronutrient, dietary fibre and salt intakes, compliance with dietary recommendations and key sources in a nationally representative sample of older adults in Ireland. In summary, this study has shown that overall intakes of macronutrients are generally sufficient. However, this population group have high intakes of total fat, saturated fat, free sugars and salt and low intakes of dietary fibre compared to recommendations. These findings are similar to those reported for older adults and other population groups across Europe [21,22,23,24,25,26,27,28,29,45,67,68].

Dietary fat is an important source of energy in the diet in addition to being an essential component of cell membranes and a precursor for many signalling molecules. Research over the past number of decades has shown that imbalances in dietary fat intake are associated with the development of many chronic diseases [62]. For example, elevated intakes of saturated fat are known to increase circulating LDL cholesterol concentrations and the risk of cardiovascular disease, while long-chain n-3 PUFAs are known to confer benefits to metabolic health and deficiency may contribute to age-related functional decline [6,69,70]. The mean intake of total fat (34%TE) in older adults in Ireland was within the reference intake range of 20–35%TE recommended by the EFSA. However, 40% of older adults had intakes above the recommended upper threshold of 35%TE [62]. Intakes of total fat among older adults in Ireland were similar to intakes in Portugal, Italy, The Netherlands, Finland, Sweden, the UK, Spain and Norway (29–35%TE) [23,24,25,27,71,72,73,74]. Mean intakes of total fat in older adults from Austria, Denmark, Germany, Hungary, Iceland, Belgium, France, Lithuania and Andorra were higher than that in adults in Ireland and above the reference intake range from the EFSA, ranging from 36 to 40%TE [21,22,75,76,77,78,79,80,81]. The key sources of total fat in older adults in Ireland were similar to the key sources among older adults in Europe (‘meat & meat products’, ‘butter & spreading fats’, ‘milk & yogurt’ and ‘cereal & cereal products’ contributing 13–20, 12–40, 12–20 and 10–22% of intakes, respectively) [18,27,73,82].

Mean saturated fat intake in older adults in Ireland was 14%TE, which exceeds the recommendation of <10%TE from the UK DoH and the Nordic Nutrition Recommendations (NNR) [49,54]. This is similar to that reported for older adults in Sweden, The UK, the Netherlands, Andorra, Finland, Norway, Denmark, Austria, Iceland and Belgium (12–17%TE) but higher than intakes in Portugal, Spain, Italy, France, Hungary and Lithuania (10–11%TE) [21,22,23,25,27,71,73,74,76,77,78,79,80]. MUFA and PUFA fat provided 12 and 6%TE in older adults in Ireland, respectively, in line with recommendations from the UK DoH and the NNR [49,54]. Mean intakes of MUFA and PUFA are similar to that reported among older adults across Europe where intakes of MUFA range from 11%TE in the Netherlands to 17%TE in Spain and Italy and intakes of PUFA range from 4%TE in Italy to 7%TE in Austria, Belgium and the Netherlands [22,23,25,73,78]. While overall total fat intakes were similar in all European countries the lowest intakes of saturated fat and highest intakes of MUFA were observed in France, Portugal, Spain and Italy, countries which are well known for their adherence to the Mediterranean diet [83]. However, despite these slight differences in intakes the key dietary sources of saturated fat, MUFA and PUFA were similar in older adults across Europe (including the older population of Ireland) with ‘meat & meat products’ and ‘butter & spreading fats’ making the greatest contributions to intakes [18,27,73,82]. Research in recent years has explored the potential to enhance the fatty acid composition of meat, which may be an effective strategy to aid in the reduction in saturated fat in population groups [84,85].

Intakes of omega-3 fatty acids were generally in line with recommendations for this age group. The mean intake of ALA in older adults (0.56%TE) was above the recommended AI of 0.5%TE [62], similar to intakes reported for other European adults of this age group (0.6–1.2%TE). Mean intakes of EPA and DHA combined (602 mg/day) in older adults in Ireland met the EFSA recommended AI of ≥250 mg/day. It has previously been reported that intakes of these essential fatty acids in Ireland are higher in older adults than the younger population [45]. Furthermore, these intakes of omega-3 fatty acids are higher than mean intakes reported for older adults in Austria (314 mg/day), the Netherlands (290 mg/day) and median intakes in France (195 mg/day) [25,78,86].

The current dietary recommendations for protein within Europe are aimed at preventing deficiencies and are based on nitrogen balance rather than achieving an optimal intake to maintain health and function [54,63]. The NNR recommend that protein provides 15–20%TE for older adults (≥65 years), while the EFSA have set an EAR of 0.66 g/kg body weight/day for all adults [54,63]. The mean protein intake in older adults in Ireland was 1.0 g/kg/body weight/day and protein provided 18%TE, in line with findings among older adults in the UK and across Europe (15–20%TE) [21,22,23,24,25,27,71,72,73,74,75,76,77,78,79,80,81]. One-third (33%) of older adults had intakes of protein below the EAR, higher than that reported in a recent systematic review of community-dwelling older adults in Western populations, which showed that 10% of adults aged 60+ years did not meet the EAR for protein (0.66 g/kg body weight/day) [26]. As the population of older adults is ever increasing, the age-associated loss of skeletal muscle mass and strength (sarcopenia) is becoming a greater concern, with recent studies suggesting that the current recommendations for protein may not be adequate for maintenance of optimal muscle function and quality of life in the older population [16,87]. The prevalence of sarcopenia in this study was 11% (data not shown—calculated using predictive equations from a European working group on consensus definition and diagnosis of sarcopenia [88]) which is lower than the prevalence in community-dwelling adults aged over 60 in other developed countries (range 1–75%) [89,90]. Nonetheless, insufficient protein intake can lead to negative protein balance, resulting in skeletal muscle atrophy, impaired muscle growth, and functional decline and this coupled with decreased muscle protein responsiveness to protein intake (anabolic resistance) in older adults highlights the possible need for increased requirements in this age group. There is good quality consistent evidence that a protein intake of 1.0–1.3 g/kg body weight/day reduces age-related muscle mass loss The PROTAGE study group and the European Society for Clinical Nutrition and Metabolism (ESPEN) expert group recommended that the protein RDA for older adults should be increased to 1–1.2 g/kg body weight/day [15,91] and the NNR have suggested an even higher protein RDA of 1.2–1.4 g/kg body weight/day [92]. The key sources of protein in older adults in Ireland are similar to that reported for older adults across Europe with ‘meat & meat products’, ‘cereal & cereal products’ and ‘milk & milk products’ being the primary dietary sources contributing on average 28–35, 17–25 and 12–18% of intakes, respectively [18,27,73,82].

While the absolute dietary requirement for glycaemic carbohydrates is not known, energy balance is the ultimate goal and so Dietary Reference Values for carbohydrates are set to meet energy needs in the context of acceptable intake levels of fat and protein [61]. The mean intake of carbohydrate in older adults in Ireland (44%TE) was approaching the reference intake range of 45–60%TE recommended by both the EFSA and the Nordic Nutrition Recommendations (NNR) [54,61]. Mean intakes of carbohydrates among older adults across Europe range from 39 to 40%TE in Iceland and Spain to 47–48%TE in Lithuania, Germany and Portugal [73,74,75,77,80].

The mean intake of free sugars in older adults in Ireland was 8%TE, which exceeds the SACN average population intake of <5%TE [50]. Furthermore, 30% of older adults had intakes exceeding the recommendation for free sugar intake from the WHO of <10%TE for individuals and 76% had intakes greater than the WHO conditional recommendation of <5%TE [51]. Although data on intakes of free sugars in population groups globally are limited; these findings are higher than that reported for Spanish adults (65–75 years) of 5%TE and lower than that reported for older adults in the UK (11%TE) and adults aged 51–69 years in the Netherlands (11%TE) [27,93,94]. However, the key dietary sources of free sugars in older adults are similar to the sources reported for older adults in Spain and the UK where ‘top-shelf’ foods such are sugars and confectionary, cereals, bakery & pastry items and non-alcoholic beverages were the key sources contributing on average 27–36, 21–33 and 10–25% of intakes, respectively [27,94].

The mean intake of dietary fibre (19 g/day) is low among older adults in Ireland compared to the AI of 25 g/day from the EFSA and the recommended average population intake from SACN of 30 g/day [50,61]. These findings are similar to that reported across Europe where mean intakes typically range from 15–23 g/day with the exception of Norway and Germany where mean intakes are 25 and 26 g/day, respectively [21,22,23,24,25,27,71,72,73,74,75,76,77,78,79,80,81]. Inadequate fibre intake can lead to impaired bowel function and constipation, which can adversely affect quality of life and it is therefore important that low intakes of fibre are addressed in older adults to ensure a high quality of life into the later years. Furthermore, the EFSA has reported that there is evidence of benefit to health associated with consumption of dietary fibre intakes greater than 25 g/day (e.g., reduced risk of coronary heart disease and type 2 diabetes and improved weight maintenance) and this evidence should be considered when developing food-based dietary guidelines [61]. The key sources of dietary fibre among older adults in Ireland were ‘bread & rolls’ (29%), ‘vegetables & vegetables dishes’ (18%) and ‘fruit & fruit juices’ (15%). However, older adults in Ireland had just 3.6 servings of fruit and vegetables per day [67], which is below the recommended servings of 5 per day [95]—similar to that reported for older adults in the UK of 3.9 servings per day with just 26% achieving 5 a day [27], which suggests that increased intakes of fruit and vegetables more in line with recommendations may help to improve intakes of dietary fibre. However, it is important to note that dietary fibre intakes in older adults are still below recommendations among populations with higher intakes of fruit and vegetables such as France, Italy and Portugal (approx. 4.5–6 servings per day) [21,74,96].

The mean intake of salt from food sources only in older adults in Ireland was 5.6 g/day (equivalent to 2236 mg/day of sodium) which is similar to intakes reported from food alone in older adults in Western Countries, including the UK, Germany, the Netherlands, Sweden, France, Austria and Denmark (5–9 g/day) [21,25,71,75,78,97,98]. However, it is estimated that 25–30% of salt intake in Irish adults (similar to other countries) is from discretionary sources [99] and so urinary salt is a more accurate reflection of true intake. Mean urinary salt excretion exceeded the maximum population target of <6 g/day for both men (11.4 g/day) and women (7.2 g/day) [55]. The key sources of salt in older adults in Ireland (‘bread & rolls’, ‘meat & meat products’ and ‘soups & sauces’) are similar to that reported for older adults in the UK (cereals, meats and miscellaneous foods contributing 21, 26 and 11% of intakes, respectively) [27].

The key strengths of this study are the nationally representative sample included in the NANS and the detailed dietary intake data with food intake data collected at the brand level. Another important strength is the use of statistical modelling to estimate the ‘usual intakes’ of nutrients, resulting in a better estimate of the true distribution of usual intakes. While food records are considered amongst the gold standard of dietary assessment, it is well acknowledged that misreporting or under reporting of energy intake is a limitation with all dietary assessment; this issue was minimised by a high-level of researcher–participant interaction (three-visits over the 4 day period) by trained nutritionists. Additionally, for macronutrients and dietary fibre, intakes were expressed as a percentage of energy intake which partially corrects this bias.

## 5. Conclusions

In conclusion, this study has shown that while intakes of macronutrients are generally sufficient in older adults in Ireland, this population group has high intakes of total fat, saturated fat, sugar and salt and low intakes of dietary fibre. Despite some possible differences in food cultures between countries (e.g., Mediterranean vs. Western diet), insufficient intakes and similar sources of these nutrients have been identified in older adults and other population groups across Europe, highlighting that a harmonised approach to improve these dietary intakes may be an effective public health strategy. Future strategies to address these nutritional issues should include the promotion of healthy food choices in line with dietary guidelines, for example the promotion of increased intakes of fruit and vegetables and the reduced intake of products high in fat and sugar. However, additional strategies may be necessary to account for practical issues associated with an ageing population such as decreased food intake, impaired sensory perception, malabsorption, declining activity and increased disability. Thus, improvements of the food supply through reformulation in terms of fat, sugar and salt could be an effective strategy to ensure intakes more in line with recommendations.

The data presented in this study contributed to the development of the updated food-based dietary guidelines for older adults in Ireland and information about the relative contributions of specific foods to nutrient intakes will be useful to both policy makers and the food industry to develop targeted dietary strategies to improve the diets of older adults.

## Figures and Tables

**Table 1:** Key characteristics (%) of the older adult sample in the National Adult Nutrition Survey compared to the Republic of Ireland (ROI) Census 2006.

	Total Population	ROI Census (2006)
	(*n* = 226)	
	%	%
**Gender**		
Men	47	44
Women	53	56
**Age group**		
65–74 years old	66	56
75+ years old	34	44
**Season of sampling**		
Winter (September–February)	54	-
Summer (March–August)	46	-
**Location**		
Urban	80	44
Rural	20	56
Social class *		
Professional workers	12	8
Managerial and technical	37	32
Non-manual	20	21
Skilled manual	21	21
Semi-skilled	10	13
Unskilled	<1	5
Education *		
Primary	30	23
Intermediate	23	20
Secondary	20	28
Tertiary	27	29

* Social class and education data from the ROI Census available only for the total population aged 15 years and older.

**Table 2:** Usual intakes of energy and fats in older adults (≥65 years) in Ireland, in the total population and by gender.

	DRV	All (*n* = 226)		Men (*n* = 106)		Women (*n* = 120)	
		Mean ± SD	Median (IQR)	Mean ± SD	Median (IQR)	Mean ± SD	Median (IQR)
Total energy (MJ)		7.4 ± 2.0	7.2 (5.9–8.7)	8.3 ± 2.0	8.1 (6.9–9.5)	6.6 ± 1.7	6.4 (5.3–7.7) *
Total energy (kcal)		1755 ± 483	1714 (1412–2059)	1969 ± 469	1936 (1637–2261)	1566 ± 412	1530 (1269–1828) *
Food energy (kcal)		1734 ± 474	1697 (1398–2036)	1938 ± 459	1908 (1613–2226)	1555 ± 411	1521 (1259–1818) *
Total fat (g)		67.7 ± 22.0	65.5 (51.9–81.3)	75.4 ± 22.1	73.5 (59.7–88.9)	60.9 ± 19.6	58.8 (46.6–73.0) *
Total fat (%TE)	RI: 20–35% TE	34.1 ± 4.6	34.0 (30.9–37.2)	34.0 ± 4.6	34.0 (30.9–37.0)	34.1 ± 4.7	34.1 (30.9–37.3)
Saturated fat (g)		27.4 ± 10.1	26.2 (20.1–33.4)	30.8 ± 10.3	29.7 (23.4–36.9)	24.4 ± 9.0	23.2 (17.8–29.8) *
Saturated fat (%TE)	<10% TE	13.8 ± 2.8	13.6 (11.8–15.6)	13.9 ± 2.8	13.8 (12.0–15.7)	13.6 ± 2.8	13.5 (11.7–15.5) *
Monounsaturated fat (g)		23.9 ± 8.0	23.0 (18.1–28.7)	26.7 ± 8.1	25.9 (20.9–31.6)	21.4 ± 7.1	20.6 (16.2–25.7) *
Monounsaturated fat (%TE)	6%	12.0 ± 1.9	11.9 (10.6–13.2)	12.0 ± 1.9	12.0 (10.7–13.2)	11.9 ± 1.9	11.8 (10.6–13.2) *
Polyunsaturated fat (g)		11.4 ± 4.2	10.8 (8.3–13.8)	12.1 ± 4.3	11.6 (9.1–14.6)	10.7 ± 3.9	10.1 (7.8–13.0) *
Polyunsaturated fat (%TE)	12%	5.7 ± 1.4	5.6 (4.7–6.6)	5.5 ± 1.3	5.4 (4.6–6.3)	6.0 ± 1.4	5.8 (4.9–6.9) *
Total n-3PUFA (g)		1.9 ± 1.2	1.5 (1.0–2.3)	2.0 ± 1.3	1.7 (1.1–2.5)	1.7 ± 1.1	1.4 (0.9–2.1) *
Total n-3PUFA (%TE)		0.93 ± 0.54	0.80 (0.56–1.16)	0.91 ± 0.53	0.79 (0.55–1.13)	0.95 ± 0.56	0.81 (0.56–1.19) *
ALA (g)		1.10 ± 0.49	1.01 (0.74–1.36)	1.11 ± 0.50	1.03 (0.76–1.37)	1.08 ± 0.49	1.00 (0.73–1.35) *
ALA (%TE)	AI: 0.5% TE	0.56 ± 0.21	0.52 (0.41–0.67)	0.50 ± 0.18	0.48 (0.37–0.60)	0.61 ± 0.22	0.57 (0.45–0.73) *
EPA (mg)		265 ± 406	141 (63.6–308)	300 ± 444	162 (74.2–345)	234 ± 366	124 (56.8–276) *
EPA (%TE)		0.14 ± 0.21	0.08 (0.03–0.16)	0.14 ± 0.20	0.08 (0.04–0.16)	0.13 ± 0.21	0.07 (0.03–0.16) *
DHA (mg)		328 ± 461	185 (85.5–391)	380 ± 528	217 (102–451)	282 ± 387	159 (75.1–340) *
DHA (%TE)		0.17 ± 0.24	0.10 (0.05–0.21)	0.18 ± 0.25	0.10 (0.05–0.21)	0.16 ± 0.23	0.09 (0.04–0.20) *
DHA ± EPA (mg)	AI: 250 mg/day	602 ± 871	335 (157–711)	726 ± 1013	415 (197–849)	493 ± 704	277 (132–591) *
DHA ± EPA (%TE)		0.31 ± 0.43	0.18 (0.09–0.37)	0.34 ± 0.46	0.20 (0.10–0.40)	0.28 ± 0.40	0.16 (0.08–0.34) *

PUFA, polyunsaturated fatty acids; ALA, α-Linolenic acid; EPA, eicosapentaenoic acid; DHA, docosahexaenoic acid; RI, recommended intake; AI, adequate intake. * Statistically different (*p* < 0.001) from that of men within the rows via Independent Samples T Tests of Mann–Whitney U Tests and adjusted for multiple testing.

**Table 3:** Usual intakes of protein, carbohydrate, sugars, dietary fibre, salt and alcohol in older adults (≥65 years) in Ireland, in the total population and by gender.

	DRV	All (*n* = 226)		Men (*n* = 106)		Women (*n* = 120)	
		Mean ± SD	Median (IQR)	Mean ± SD	Median (IQR)	Mean ± SD	Median (IQR)
Protein (g)		77.6 ± 19.8	76.0 (63.6–90.2)	85.8 ± 19.3	84.6 (72.2–97.9)	70.4 ± 17.3	68.9 (57.9–81.4) *
Protein (%TE)		18.2 ± 2.8	18.0 (16.2–20.0)	18.0 ± 2.7	17.9 (16.1–19.7)	18.4 ± 2.8	18.2 (16.4–20.2) *
Protein (g/kg body weight)	EAR: 0.66 g/kg body weight	1.0 ± 0.1	0.9 (0.6–1.3)	0.9 ± 0.1	0.9 (0.5–1.2)	1.0 ± 0.1	0.9 (0.6–1.3) *
Carbohydrate (g)		208 ± 62.7	203 (163–247)	227 ± 62.4	223 (183–266)	190 ± 57.6	185 (149–227) *
Carbohydrate (%TE)	RI: 45–60% TE	44.4 ± 5.5	44.4 (40.6–48.1)	43.2 ± 5.3	43.2 (39.6–46.8)	45.5 ± 5.4*	45.4 (41.8–49.2)
Total sugars (g)		86.2 ± 36.5	81.2 (59.4–107.8)	89.5 ± 37.0	84.9 (62.7–111.0)	83.3 ± 35.8	78.2 (56.9–104.5) *
Total sugars (%TE)		18.2 ± 5.3	17.9 (14.4–21.6)	16.8 ± 4.9	16.4 (13.3–19.8)	19.5 ± 5.3	19.2 (15.7–23.0) *
Added sugars (g)		37.4 ± 25.0	31.8 (18.9–50.0)	39.7 ± 25.8	34.4 (20.8–52.5)	35.3 ± 24.1	29.8 (17.5–47.5) *
Added sugars (%TE)		7.5 ± 4.2	6.8 (4.4–9.8)	7.2 ± 4.0	6.5 (4.3–9.4)	7.7 ± 4.2	7.0 (4.6–10.1) *
Free sugars (g)		41.0 ± 26.4	35.5 (21.5–54.8)	43.0 ± 27.0	37.7 (23.2–56.9)	39.2 ± 25.7	33.7 (20.2–52.6) *
Free sugars (%TE)	<5% <10%	8.3 ± 4.4	7.6 (5.1–10.8)	7.8 ± 4.2	7.2 (4.8–10.2)	8.7 ± 4.5	8.0 (5.4–11.3) *
Dietary fibre (g)	AI: >25 g/day	19.0 ± 6.7	18.3 (14.2–23.2)	19.6 ± 6.7	18.9 (14.7–23.6)	18.5 ± 6.6	17.8 (13.7–22.6) *
Dietary fibre (g/10 MJ)		26.6 ± 7.2	26.0 (21.4–31.1)	24.3 ± 6.4	23.8 (19.8–28.3)	28.6 ± 7.2	28.0 (23.4–33.2) *
Sodium (mg)		2236 ± 653	2181 (1769–2645)	2562 ± 619	2521 (2124–2950)	1948 ± 536	1901 (1561–2289) *
Salt equivalent (g)	<6 g/day	5.6 ± 1.6	5.4 (4.4–6.6)	6.4 ± 1.6	6.3 (5.3–7.4)	4.9 ± 1.3	4.7 (3.9–5.7) *
Alcohol (g)		4.5 ± 16.3	1.0 (0.3–3.2)	7.1 ± 20.8	1.9 (0.6–5.7)	2.1 ± 10.5	0.5 (0.2–1.7) *

EAR, estimated average requirement; RI, recommended intake; AI, adequate intake. * Statistically different (*p* < 0.001) from that of men within the rows via Independent Samples T Tests of Mann–Whitney U Tests and adjusted for multiple testing.

## Data Availability

The data presented in this study are available from the corresponding author upon reasonable request.

## Supplementary Materials

The following are available online at https://www.mdpi.com/2072-6643/13/3/876/s1, Table S1: Usual intakes of energy and fats in older adults (≥65 years) in Ireland by age group, Table S2: Usual intakes of protein, carbohydrates, sugars, dietary fibre, salt and alcohol in older adults (≥65 years) in Ireland by age group.
