# Supplementary material for: Energy, Macronutrients, Dietary Fibre and Salt Intakes in Older Adults in Ireland: Key Sources and Compliance with Recommendations
Source: Nutrients. 2021 Mar 8;13(3):876. doi: 10.3390/nu13030876 (PMC8001736; doi:10.3390/nu13030876)
Supplement: Supplementary file 1 [file nutrients-13-00876-s001.pdf]

Supplementary Table 1. Usual intakes of energy and fats in older adults (≥65 years) in Ireland by age group

	DRV	65-75 year olds (*n* 149)		75+ year olds (*n* 77)	
		**Mean ± SD**	**Median (IQR)**	**Mean ± SD**	**Median (IQR)**
Total energy (MJ)		7.4 ± 2.0	7.2 (6.0 – 8.7)	7.4 ± 2.0	7.2 (5.9 – 8.6)
Total energy (kcal)		1759 ± 484	1714 (1415 - 2062)	1748 ± 481	1713 (1405 - 2055)
Food Energy (kcal)		1738 ± 475	1697 (1402 – 2038)	1728 ± 473	1697 (1393 – 2031)
Total Fat (g)		67.8 ± 22.1	65.5 (52.0 – 81.4)	67.5 ± 21.9	65.6 (51.7 – 81.0)
Total fat (%TE)	RI: 20 - 35% TE	34.0 ± 4.6	34.0 (30.9 – 37.2)	34.1 ± 4.6	34.1 (30.9 - 37.3)
Saturated fat (g)		27.4 ± 10.2	26.2 (20.1 – 33.5)	27.3 ± 10.1	26.2 (20.0 – 33.4)
Saturated fat (%TE)	<10% TE	13.8 ± 2.8	13.6 (11.8 - 15.6)	13.8 ± 2.8	13.7 (11.8 - 15.6)
Monounsaturated fat (g)		23.9 ± 8.0	23.0 (18.2 – 28.8)	23.8 ± 8.0	23.0 (18.0 - 28.7)
Monounsaturated fat (%TE)	6%	11.9 ± 1.9	11.9 (10.6 - 13.2)	12.0 ± 1.9	11.9 (10.7 - 13.2)
Polyunsaturated fat (g)		11.4 ± 4.2	10.8 (8.3 – 13.8)	11.3 ± 4.1	10.8 (8.3 – 13.8)
Polyunsaturated fat (%TE)	12%	5.7 ± 1.4	5.6 (4.7 - 6.6)	5.8 ± 1.4	5.6 (4.8 – 6.6)
Total n-3PUFA (g)		1.9 ± 1.2	1.5 (1.0 - 2.3)	1.8 ± 1.2	1.5 (1.0 - 2.3)
Total n-3PUFA (%TE)		0.93 ± 0.55	0.80 (0.55 - 1.16)	0.93 ± 0.53	0.81 (0.56 - 1.17)
ALA (g)		1.10 ± 0.50	1.01 (0.74 - 1.36)	1.10 ± 0.49	1.02 (0.74 - 1.36)
ALA (%TE)	AI: 0.5% TE	0.56 ± 0.21	0.52 (0.41 - 0.67)	0.56 ± 0.21	0.53 (0.41 - 0.68)
EPA (mg)		265 ± 410	142 (64 - 307)	264 ± 397	139 (64 - 310)
EPA (%TE)		0.14 ± 0.21	0.08 (0.03 - 0.16)	0.15 ± 0.20	0.07 (0.04 - 0.16)
DHA (mg)		327 ± 460	187 (86 - 394)	328 ± 463	181 (85 - 385)
DHA (%TE)		0.17 ± 0.24	0.10 (0.05 - 0.21)	0.17 ± 0.24	0.10 (0.05 - 0.21)
DHA ± EPA (mg)	AI: 250mg/d	603 ± 874	338 (158 - 711)	600 ± 863	327 (155 - 710)
DHA ± EPA (%TE)		0.31 ± 0.43	0.18 (0.09 - 0.37)	0.31 ± 0.42	0.18 (0.09 - 0.37)

PUFA, polyunsaturated fatty acids; ALA, α-Linolenic acid; EPA, eicosapentaenoic acid; DHA, docosahexaenoic acid

No statistical differences (P < 0.001) were noted between age groups for any nutrient examined

Supplementary Table 2. Usual intakes of protein, carbohydrate, sugars, dietary fibre, salt and alcohol in older adults (≥65 years) in Ireland by age group

	DRV	65-75 year olds (*n* 149)		75+ year olds (*n* 77)	
		**Mean ± SD**	**Median (IQR)**	**Mean ± SD**	**Median (IQR)**
Protein (g)		77.8 ± 19.9	76.0 (63.7 - 90.2)	77.4 ± 19.8	76.0 (63.3 – 90.0)
Protein (%TE)		18.2 ± 2.8	18.0 (16.2 – 20.0)	18.2 ± 2.8	18.1 (16.3 - 20.0)
Protein (g/kg body weight)	AR: 0.66g/kg body wt	1.0 ± 0.6	0.9 (0.6 - 1.3)	1.0 ± 0.5	0.9 (0.6 - 1.3)
Carbohydrate (g)		208 ± 62.8	203 (164 - 248)	207 ± 62.4	203 (163 - 247)
Carbohydrate (%TE)	RI: 45 - 60% TE	44.4 ± 5.5	44.3 (40.6 – 48.0)	44.5 ± 5.5	44.5 (40.8 - 48.3)
Total sugars (g)		86.2 ± 36.6	80.9 (59.5 - 108)	86.3 ± 36.3	81.8 (59.4 - 108)
Total sugars (%TE)		18.2 ± 5.3	17.8 (14.3 – 21.5)	18.4 ± 5.3	18.0 (14.6 – 21.8)
Added sugars (g)		37.3 ± 25.1	31.6 (18.9 – 50.0)	37.4 ± 24.8	32.2 (18.9 – 50.0)
Added sugars (%TE)		7.5 ± 4.2	6.7 (4.4 – 9.8)	7.5 ± 4.1	6.9 (4.5 – 9.9)
Free sugars (g)		41.0 ± 26.5	35.2 (21.5 – 54.8)	41.1 ± 26.2	25.9 (21.6 – 54.9)
Free sugars (%TE)	< 5% < 10%	8.3 ± 4.4	7.6 (5.0 – 10.8)	8.4 ± 4.3	7.7 (5.2 – 10.9)
Dietary Fibre (g)	AI: >25g/d	19.0 ± 6.7	18.2 (14.1 - 23.1)	19.0 ± 6.7	18.4 (14.2 – 23.2)
Dietary Fibre (g/10MJ)		26.5 ± 7.2	25.9 (21.3 – 31.0)	26.8 ± 7.1	26.2 (21.6 – 31.3)
Sodium (mg)		2242 ± 654	2183 (1776 - 2654)	2224 ± 651	2179 (1756 - 2634)
Salt equivalent (g)	<6g/d	5.6 ± 1.6	5.4 (4.4 – 6.6)	5.6 ± 1.6	5.4 (4.4 – 6.6)
Alcohol (g)		4.5 ± 16.8	1.0 (0.3 – 3.3)	4.3 ± 15.2	1.0 (0.3 – 3.2)

No statistical differences (P < 0.001) were noted between age groups for any nutrient examined
